# CD99 Expression and Prognostic Impact in Glioblastoma: A Single-Center Cohort Study

**DOI:** 10.3390/cells13070597

**Published:** 2024-03-29

**Authors:** Andrea Rocca, Fabiola Giudici, Carmine Antonio Donofrio, Cristina Bottin, Maurizio Pinamonti, Benvenuto Ferrari, Francesco Schettini, Estela Pineda, Stefano Panni, Marika Cominetti, Patrizia D’Auria, Simonetta Bianchini, Elena Varotti, Marco Ungari, Stefano Ciccarelli, Marzia Filippini, Sarah Brenna, Valentina Fiori, Tomas Di Mambro, Angelo Sparti, Mauro Magnani, Fabrizio Zanconati, Daniele Generali, Antonio Fioravanti

**Affiliations:** 1 Department of Medical, Surgical and Health Sciences, University of Trieste, 34147 Trieste, Italy; 2 Cancer Epidemiology Unit, Centro di Riferimento Oncologico di Aviano (CRO) IRCCS, 33081 Aviano, Italy; 3 Neurosurgery, ASST Cremona, Viale Concordia 1, 26100 Cremona, Italy; 4 Division of Biology and Genetics, Department of Molecular and Translational Medicine, University of Brescia, Viale Europa 11, 25123 Brescia, Italy; 5 Breast and Brain Unit, ASST Cremona, Viale Concordia 1, 26100 Cremona, Italy; 6 Translational Genomics and Targeted Therapies in Solid Tumors Group, August Pi i Sunyer Biomedical Research Institute (IDIBAPS), C. Villaroel 170, 08036 Barcelona, Spain; 7 Medical Oncology Department, Hospital Clínic of Barcelona, 08036 Barcelona, Spain; 8 Facultat de Medicina i Ciències de la Salut, Universitat de Barcelona, 08036 Barcelona, Spain; 9 Pathology Unit, ASST Cremona, Viale Concordia 1, 26100 Cremona, Italy; 10 Radiotherapy Unit, ASST Cremona, Viale Concordia 1, 26100 Cremona, Italy; 11 Diatheva srl, 61030 Cartoceto, Italy; 12 Department of Biomolecular Sciences, University of Urbino Carlo Bo, 61029 Urbino, Italy

**Keywords:** glioblastoma, prognostic factor, CD99, quantitative real-time polymerase chain reaction, immunohistochemistry

## Abstract

Glioblastoma is the most frequent and aggressive brain tumor in adults. This study aims to evaluate the expression and prognostic impact of CD99, a membrane glycoprotein involved in cellular migration and invasion. In a cohort of patients with glioblastoma treated with surgery, radiotherapy and temozolomide, we retrospectively analyzed tumor expression of CD99 by immunohistochemistry (IHC) and by quantitative real-time polymerase chain reaction (qRT-PCR) for both the wild type (CD99wt) and the truncated (CD99sh) isoforms. The impact on overall survival (OS) was assessed with the Kaplan–Meier method and log-rank test and by multivariable Cox regression. Forty-six patients with glioblastoma entered this study. Immunohistochemical expression of CD99 was present in 83%. Only the CD99wt isoform was detected by qRT-PCR and was significantly correlated with CD99 expression evaluated by IHC (rho = 0.309, *p* = 0.037). CD99 expression was not associated with OS, regardless of the assessment methodology used (*p* = 0.61 for qRT-PCR and *p* = 0.73 for IHC). In an exploratory analysis of The Cancer Genome Atlas, casuistry of glioblastomas CD99 expression was not associated with OS nor with progression-free survival. This study confirms a high expression of CD99 in glioblastoma but does not show any significant impact on survival. Further preclinical studies are needed to define its role as a therapeutic target in glioblastoma.

## 1. Introduction

Glioblastoma is the most frequent and aggressive primary tumor of the central nervous system (CNS) in adults, with an annual age-adjusted incidence rate of 3.6 per 100.000 in the United States and a 5-year overall survival (OS) rate of 6.9% among all comers [1,2]. Patients receiving standard treatment with maximum safe surgery followed by radiotherapy and adjuvant temozolomide have a median OS of about 15 months, which reduces to 8 months in those unsuitable for standard therapy [2,3,4].

Numerous efforts have been made to improve these outcomes, ranging from optimizing radiotherapy delivery [5] and intensifying adjuvant chemotherapy [6] to exposing tumors to low-intensity alternating electric fields (Tumor-Treating Fields, TTFs) [7]. TTFs significantly improved progression-free and overall survival (OS) when added to adjuvant temozolomide, but they are still unavailable in most countries. Additionally, various new drugs have been tested in advanced disease, including antiangiogenic agents and immune checkpoint inhibitors, with limited efficacy [3].

Alongside exploring new drugs, research has focused on understanding the molecular alterations underlying glioblastoma. Transcriptomics studies have identified different subtypes of glioblastoma, reflecting distinct stages of neurogenesis and neural cell types. These subtypes show genomic abnormalities in distinct signaling pathways and present with differential prognostic impact and varying degrees of benefit from standard treatment [8,9]. Integrated multi-omics analyses further refined the characterization of glioblastoma subtypes [10,11,12] and their key molecular alterations, with the final aim of detecting potential new therapeutic targets.

CD99 is a glycosylated transmembrane protein encoded by the pseudoautosomal gene *MIC2*. It is expressed at low levels in most human cells and is involved in crucial cellular functions, such as apoptosis, adhesion, differentiation, and migration [13]. CD99 primarily functions by creating homophilic interactions with CD99 present on adjacent cells, regardless whether they are of the same or different types. Although the existence of CD99 ligands on the membrane of certain cell types is hypothesized, they are still incompletely characterized [14,15]. CD99’s role is particularly important in hematopoiesis, lymphocyte and granulocyte functioning, and neural cell differentiation. According to The Human Protein Atlas (HPA), immunohistochemical expression of CD99 in normal tissues is higher in the brain, proximal digestive tract, pancreas, organs of the reproductive system, bone marrow, and lymphoid tissues. Single-cell mRNA expression data show higher levels in melanocytes, Leydig cells, NK-cells, Langerhans cells, and fibroblasts [16].

CD99 is commonly overexpressed in some tumor types, particularly in Ewing sarcoma, acute leukemias/myelodysplastic syndromes, and malignant gliomas, and more sporadically in other tumors, while it can be downregulated in other neoplasms [13]. It can therefore function as an oncogene or as a tumor suppressor in different contexts. Preclinical experiments have shown that silencing CD99 in tumors where it is overexpressed or inducing its expression when downregulated leads to a reversal of the malignant phenotype, identifying CD99 as a potentially relevant therapeutic target [13].

CD99 exists in two isoforms resulting from alternative splicing: the wild-type full-length CD99 type I (CD99wt) and the truncated form CD99 type II (CD99sh), lacking most of the intracellular domain. These isoforms have different and sometimes opposite functions, exhibit different expressions in different cell types, and can dimerize to form heterodimers. In tumors, they have been reported to exert opposite effects, with CD99wt inhibiting and CD99sh favoring cell migration, invasion, and metastasis [17]. However, their effects may differ in different neoplasms [18].

In a study of cDNA microarrays, CD99 emerged as one of the genes differentially expressed between glioblastoma and normal encephalic tissue and was part of a selection of 31 genes encoding membrane proteins that represent potential targets for cellular or antibody-based immunological therapies [19]. Further analyses of the glioblastoma transcriptome, reconstructing the network of critical genes which were differentially expressed between glioblastoma and normal tissue by means of Bayesian network analysis, identified a set of ten genes whose expression levels are sufficient to predict the probability of developing glioblastoma, including CD99 [20]. Preclinical and translational studies have confirmed the overexpression of CD99 in glioblastoma and highlighted its role in promoting glioblastoma cell migration and invasion [21,22,23,24,25,26].

The purpose of this retrospective study is to investigate the expression levels and prognostic impact of CD99 in a single-center cohort of patients with glioblastoma, supporting its relevance as a promising therapeutic target. An in silico confirmatory analysis in The Cancer Genome Atlas (TCGA) dataset [27] was then conducted.

## 2. Materials and Methods

### 2.1. Patients’ Characteristics

We retrospectively collected formalin-fixed paraffin-embedded (FFPE) tumor samples from a cohort of patients who underwent surgical resection of a brain tumor with histological diagnosis of glioblastoma, according to the 2021 World Health Organization (WHO) Classification of Tumors of the CNS. The patients had been treated at the Neurosurgery Division of the Hospital of Cremona, Italy, between 2018 and 2020. We identified 72 patients and further details for this cohort can be found in a recently published paper [28]. Of these patients, 46 cases had available pathological material to analyze the immunohistochemical expression of CD99, as well as the expression levels of the two CD99 isoforms using quantitative Real-time Polymerase Chain Reaction (qRT-PCR). Demographic, clinical, and survival data were retrieved from clinical charts. This study was approved by the Ethics Committee of the Territorial Social Health Service of Cremona (protocol number 32219, dated 2 October 2019).

### 2.2. Immunohistochemistry

FFPE blocks were cut into serial 3 μm thick slices with a microtome. IHC for CD99 was performed with a Ventana Benchmark Ultra immunostaining automated system. Immunostaining was performed with the CONFIRM anti-CD99 (O13) Mouse Monoclonal Primary Antibody (Ventana Medical Systems, Tucson, AZ, USA). The staining was performed using the OptiView DAB IHC Detection Kit (Ventana Medical Systems, Tucson, AZ, USA), according to the following procedure: first the slides were deparaffinized at 72°, then treated with ULTRA Cell Conditioning (ULTRA CC1) (Ventana Medical Systems, Tucson, AZ, USA) for 56 min at 95 °C in order to reveal the antigen. Afterward the endogenous peroxidase was inhibited with OptiView Peroxidase Inhibitor (H202 3.0%)(Ventana Medical Systems, Tucson, AZ, USA), followed by the incubation of the antigen with the CONFIRM anti-CD99 (O13) Mouse Monoclonal Primary Antibody (Ventana Medical Systems, Tucson, AZ, USA) for 24 min at 36 °C. Then, a cocktail of HQ antibody was added (OptiView HQ Universal Linker, Ventana Medical Systems, Tucson, AZ, USA) for 8 min as well as the OptiView HRP Multimer (Ventana Medical Systems, Tucson, AZ, USA) for another 8 min. At the end the hematoxylin II (Ventana Medical Systems, Tucson, AZ, USA), counterstain was added for 12 min followed by the bluing (Ventana Medical Systems, Tucson, AZ, USA) for 4 min. At the end of the automated immunostaining, the slides were washed and dehydrated using a gradient of ethylic alcohol and xylene and finally mounted with CV ULTRA mounting medium (Leica Biosystem Richmond, Richmond, VA, USA).

**Figure 1:**
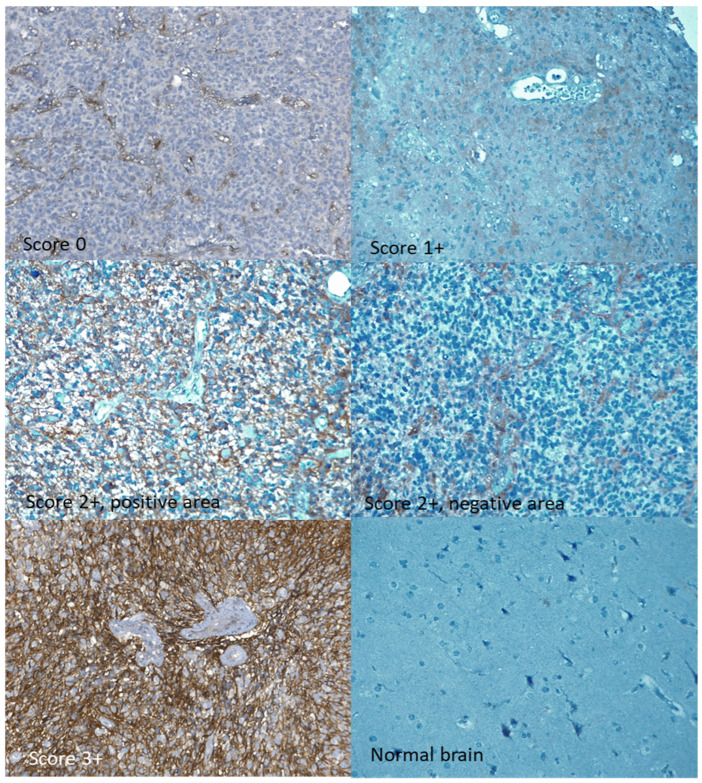
Immunohistochemical staining for CD99. All expression classes of CD99 are represented: 0, 1+, 2+ (both a positive and a negative area from the same sample), 3+. Original magnification 20×.

Immunohistochemical staining was analyzed by one pathologist who was blinded to the related clinical information. The expression patterns were divided semi-quantitatively into four categories (0, 1+, 2+, and 3+), similarly to what is performed in HER2 staining interpretation. A score 0 (negative) was given to cases in which no expression of CD99 was evident in tumor cells. Score 1+ was reserved for cases with weak and focal expression, i.e., limited to less than 10% of tumor cells. Score 2+ was attributed to cases in which there was a non-homogeneous expression pattern of CD99, with the coexistence of negative areas and areas stained with variable intensity, from weak to moderate or intense, in which the staining of moderate or intense intensity did not exceed 50% of neoplastic cells. To assign a 3+ score, moderate or intense staining had to be present in at least 50% of the neoplastic cells. (Figure 1).

### 2.3. Quantitative Real-Time PCR

Total RNA was extracted from FFPE sections using the AllPrep DNA/RNA FFPE kit (QIAGEN, Hilden, Germany); quality and concentration assessments were performed. Complementary DNA (cDNA) was obtained starting form 500 ng of total RNA by reverse transcription, using SuperScript IV reverse transcriptase, RNase inhibitor, random oligonucleotides, according to the manufacturer’s recommendations (Thermo Fisher Scientific, Waltham, MA, USA) in a total volume of 25 µL. After treatment with 1 U RNase H (Thermo Fisher Scientific, Waltham, MA, USA) at 37 °C for 20 min, cDNA was stored at −20 °C for subsequent analysis.

The relative qRT-PCR for CD99wt and CD99sh quantitation was performed on Applied Biosystems 7500 apparatus (Thermo Fisher Scientific, Waltham, MA, USA) using primers and probe elsewhere described [29]: 2 isoform-specific CD99 reverse primer for the CD99 wild-type (CD99_ wild-type: 5′-ATGTCCACCTCCCCTTGTT-3′) and short (CD99_Sh: 5′-TGCTCACCCCTAGGTCTT-3′) isoforms, and a common forward primer (CD99 Fw: 5′-CTGGAGCCATCTCTAGCTT-3′) and probe (5′-(FAM)TTCTTTTTCTGGTAAGCA-3′). *GAPDH* was used as reference gene for normalization and was amplified in a multiplex reaction with CD99 using primers and probe described in [30]: GAPDH Up: 5′-ATGGGTGTGAACCATGAGAA-3′, GAPDH Low: 5′-GTGCTAAGCAGTTGGTGGTG-3′, and the probe 5′-(VIC) CCTCAAGATCATCAGCAATGCCTCC-3′. The reaction mixture of 25 μL contained 5× PerfeCTa Multiplex qPCR ToughMix (QuantaBio, Beverly, MA, USA), 200 nmol/L of each primer and probe, and 5 μL of cDNA.

Amplification conditions were 3 min at 95 °C followed by 45 cycles at 95 °C for 15 s, and at 50 °C for 1 min. Single product amplification was confirmed by analyzing its dissociation curve. The amplification efficiencies were calculated using serial cDNA dilutions. The 2^−ddCt^ equation was applied in the calculation of the CD99 relative gene expression versus the lowest expressing samples used as calibrator (where dCt = [mean Ct of CD99] − [mean Ct of GAPDH] and ddCt = [dCt sample x − dCt lowest expressing sample]).

### 2.4. Statistical Analysis

All statistical analyses were performed with the use of R-software (version 4.2.3) [31]. Data were summarized by the median, interquartile range (IQR) reporting the first and the third quartiles for age, and by means of absolute frequencies and percentages for categorical variables. Graphical illustration of the correlation between IHC and qRT-PCR CD99 quantification is shown as a scatterplot. Spearman’s rank correlation coefficient (rho) was calculated as a measure of the strength and direction of the relationship between the two variables. The association between CD99wt values as continuous variable and categorical ones was tested by means of non-parametric Wilcoxon–Mann Whitney test or Kruskal–Wallis test, when appropriate. OS was calculated from the date of surgery to the date of death from any cause. Alive patients were censored at the date of the last follow-up. OS functions were estimated using the Kaplan–Meier method, and the log-rank test was used to assess differences between groups. Associations between CD99 and overall survival were assessed by multivariable Cox proportional hazard models adjusted for known risk factors (age, type of resection, and MGMT promoter methylation). *p* values ≤ 0.05 were considered statistically significant.

TCGA data were obtained through cBioPortal [32,33] and patients were separated into groups above or below the median of the relative transcript abundance levels (mRNA expression z-scores relative to all samples) of CD99. Differences in OS and progression-free survival (PFS) between the two groups were evaluated by the log rank test, considering q values derived from the Benjamini–Hochberg false discovery rate correction procedure to assess statistical significance.

## 3. Results

### 3.1. Patient Population

Forty-six patients with sufficient archival pathological material were included in this study and analyzed. All had a pathological diagnosis of glioblastoma (isocitrate dehydrogenase 1/2-wildtype) according to the 2021 WHO Classification of Tumors of the Central Nervous System. Median age was 65.2 years (IQR: 59.3–74.5). Fifty-nine percent of the patients were males and forty-one percent females. All had been treated with maximum safe surgery followed by radiotherapy with concurrent and adjuvant temozolomide. Most patients underwent surgical resection of a primary tumor, while five patients (10.9%) had resection for tumor relapse. The types of surgery included complete (gross total) resection in 11 cases (23.9%), partial resection in 14 cases (30.4%), and biopsy only in 15 cases (32.6%). Multifocality was observed in 8 patients (17.4%), and MGMT promoter methylation was present in 37% of the cases. Patients and tumor characteristics are fully reported in Table 1.

### 3.2. CD99 Expression and Prognostic Impact

**Figure 2:**
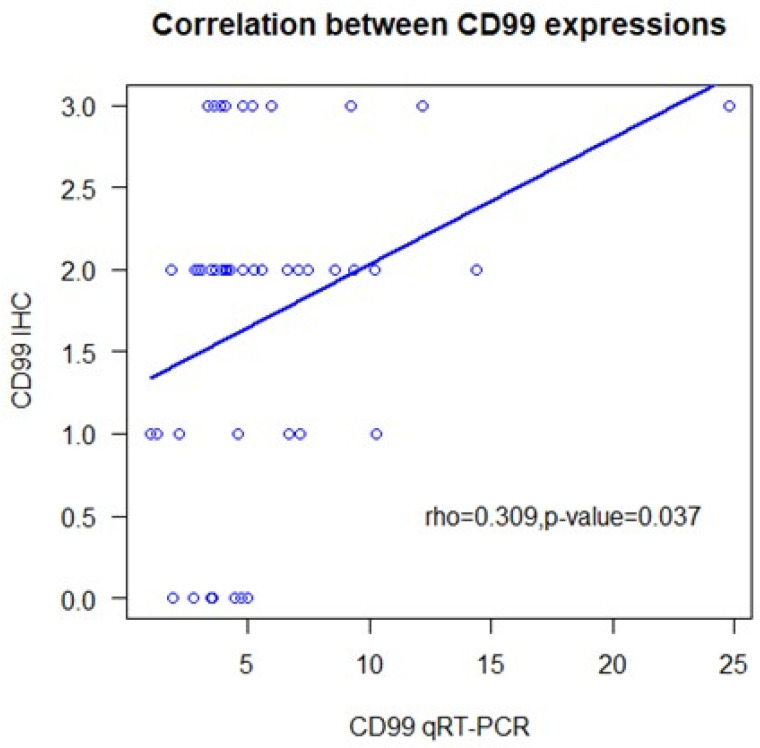
Correlation of quantitative real-time polymerase chain reaction analyses for CD99 with semi-quantitative immunohistochemistry. IHC: immunohistochemistry; qRT-PCR: quantitative real-time reverse transcriptase polymerase chain reaction.

CD99 expression, assessed by IHC, was absent in 17% of the tumors and present, at varying intensities, in 83%. Specifically, a 3+ expression score was registered in 22% of the cases, while 2+ and 1+ scores were present in 44% and 17%, respectively (Table 1). Some samples of normal brain tissue were also analyzed with IHC, with substantially negative results or with mild and focal expression in individual elements (Figure 1). qRT-PCR analysis identified the expression of the full-length CD99wt isoform, with no expression of CD99sh. CD99wt expression levels quantified by qRT-PCR were moderately correlated with CD99 expression levels assessed by IHC (rho = 0.309, 95% CI: 0.02–0.55, *p* = 0.037) (Figure 2).

Age (*p* = 0.538), sex (*p* = 0.390), and type of tumor (primary versus relapse) (*p* = 0.251) did not correlate with CD99wt levels. Higher values of CD99wt were observed in tumors that underwent complete resection (median = 6.73), compared to partial (median = 4.36) or biopsy (median = 3.51) resections (*p* = 0.011).

At univariate analysis, none of the variables were significantly associated with OS (Table 1). Younger patients experienced a numerically better survival, which did not reach statistical significance (*p* = 0.094). The 12-month overall survival rate was 20% in patients undergoing biopsy alone, toward about 50% in those undergoing partial or complete tumor resection, but also this difference is not formally significant (*p* = 0.057).

**Figure 3:**
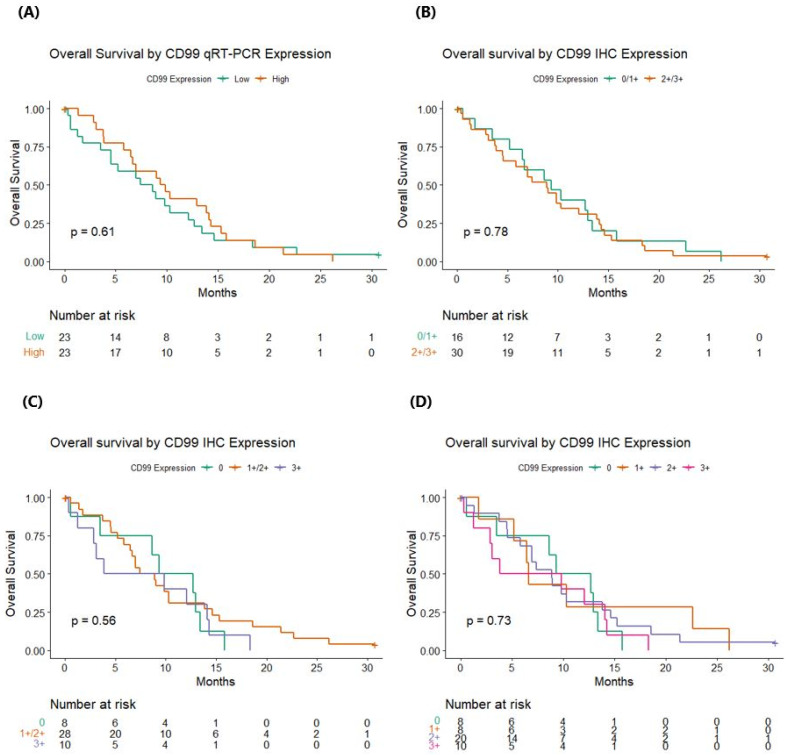
Overall survival according to CD99 status by quantitative real-time polymerase chain reaction analyses and immunohistochemistry. (**A**) Overall survival by CD99 qRT-PCR expression; (**B**) overall survival by CD99 IHC expression, considering two levels (0/1+ vs. 2+/3+); (**C**) overall survival by CD99 IHC expression, considering three levels (0 vs. 1+/2+ vs. 3+); (**D**) overall survival by CD99 IHC expression, considering four levels (0 vs. 1+ vs. 2+ vs. 3+). qRT-PCR: quantitative real-time polymerase chain reaction; IHC: immunohistochemistry. *p* values are referred to log-rank test.

CD99wt expression levels assessed by qRT-PCR and categorized according to the median value were not associated with OS (*p* = 0.607). Similarly, the expression of CD99 assessed by IHC did not significantly impact on survival, regardless of whether two (0/1+ vs. 2+/3+, *p* = 0.78), three (0 vs. 1+/2+ vs. 3+, *p* = 0.56), or four classes of expression levels (0 vs. 1+ vs. 2+ vs. 3+, *p* = 0.73) were considered (Figure 3). When considering the four levels of IHC expression, no clear trend of OS emerges.

Separate multivariate models were constructed for CD99 assessed either by IHC or qRT-PCR, adjusting for age, type of surgical resection, and MGMT promoter methylation status (Table 2). CD99 was never found to be associated with OS, while age (*p* = 0.042 in the Cox model with CD99 IHC, *p* = 0.054 in the Cox model with CD99wt qRT-PCR) and the type of surgical resection (*p* = 0.047 in the Cox model with CD99 IHC, *p* = 0.049 in the Cox model with CD99wt qRT-PCR) showed a strong prognostic impact.

### 3.3. CD99 in the TCGA Glioblastoma Dataset

**Figure 4:**
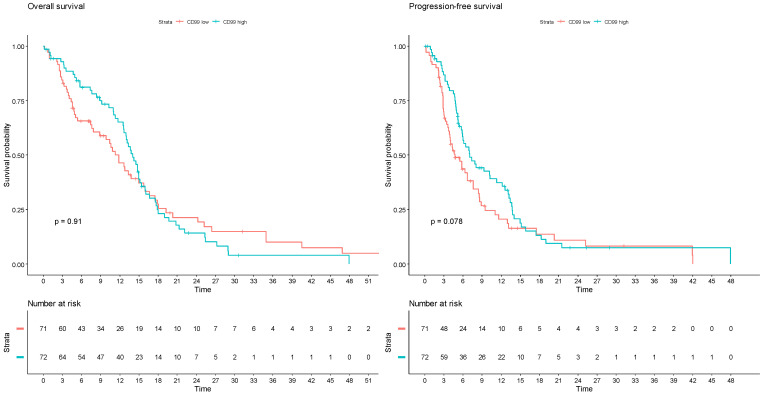
Survival analyses according to CD99 mRNA expression z-scores relative to all samples in The Cancer Genome Atlas glioblastoma dataset (PanCancer Atlas). Patients are divided into two groups, with CD99 transcript abundance levels above (CD99 high) or below (CD99 low) the median. Left: overall survival; right: progression-free survival.

In an exploratory analysis of the TCGA glioblastoma database, considering 143 patients with available genomic and transcriptomic data and IDH1/2 wildtype tumors, the expression levels of CD99 were not significantly associated with OS (log-rank test *p* = 0.912) nor with PFS (*p* = 0.0785; q-value = 0.235) (Figure 4).

## 4. Discussion

In this single-center retrospective analysis, we assessed the expression of CD99, both by IHC and by qRT-PCR, in glioblastoma tumor samples and evaluated its association with OS. An overexpression of CD99, with immunohistochemical staining score 3+, was found in 22% of the tumors, and a lower expression, corresponding to 1+ or 2+ scores, in 61%. No expression of CD99, or mild and focal expression in individual cells, was found in normal brain tissue samples. CD99, whether quantified by qRT-PCR or assessed semi-quantitatively by IHC, was not associated with OS. An analysis of the glioblastoma TCGA casuistry, although not directly comparable with our casuistry, confirms a lack of association of CD99 mRNA levels with OS and PFS.

The expression of CD99 in glioblastoma has been assessed in multiple studies [21,22,23,24,25,26], but its prognostic impact has been rarely investigated, with no evidence of association with OS [22]. CD99 is expressed at higher levels in glioblastoma compared to normal brain or lower-grade gliomas [21,22,23,26]. A prognostic impact emerged only when considering gliomas of all grades [26]. Nonetheless, CD99 is involved in cell invasiveness and migration. It may therefore play an important role in the typical tendency of glioblastoma to microscopically infiltrate the adjacent normal brain tissue, resulting in frequent residual microscopic disease even after apparently radical surgery. For these reasons, CD99 is a biomarker potentially associated with disease-free and overall survival. Given the paucity of data on its prognostic impact, this is worthy of further evaluation.

Most studies have shown exclusive expression of the full-length CD99wt isoform [21,22,23], but expression of the truncated CD99sh in a minority of cases has also been reported [25]. The role of the two isoforms of CD99 in different normal and neoplastic cellular contexts has been only partially explored, since, for instance, antibodies directed against the extracellular domain of the molecule, used in IHC studies, do not discriminate between the two isoforms [18]. In our cohort, CD99 was exclusively expressed as CD99wt. We found a significant positive correlation between CD99wt expression levels assessed by qRT-PCR and a semi-quantitative evaluation of CD99 expression by IHC.

Various preclinical studies have investigated the role of CD99 in glioblastoma pathogenesis and progression. Silencing CD99wt expression by siRNA in glioma cell lines led to reduced migration and invasiveness, without affecting cell viability and proliferation [21]. Orthotopic xenografts of glioma cell lines overexpressing CD99 developed tumors with wider spread and indistinct margins and had significantly reduced survival compared with xenografts not expressing CD99. CD99 overexpression led to decreased Rac and increased Rho activity, raising the proportion of amoeboid-type cells associated with enhanced cellular migration [21]. Another study exploring genes highly expressed in placenta and potentially related to an invasive phenotype found that CD99 exhibited the highest relative mRNA expression in glioblastoma compared to normal brain tissue and its expression was associated with larger, multilobar tumor extension. However, this study did not find an association between CD99 expression levels and patient prognosis [22]. CD99wt expression was also found to be higher in the classical and mesenchymal glioblastoma subtypes than in the proneural subtype. It was further associated with the expression of genes involved in actin dynamics, subtending the formation of focal adhesions and of lamellipodia/filopodia present on the leading edge of mobile cells [23]. Furthermore, CD99 has been implicated in cuproptosis, a form of copper-induced regulated cell death, through its crosstalk with the vascular endothelial growth factor pathway [34]. In another study, a high CD99 expression was associated with response to antiangiogenic therapy in patients with recurrent glioblastoma [35]. A recent bioinformatic analysis from multiple gliomas datasets highlighted an association of CD99 overexpression with tumor hypoxia, angiogenesis, epithelial-mesenchymal transition, metabolic reprogramming, and an immunosuppressive microenvironment dominated by M2 tumor-associated macrophages [26]. These represent potential mechanisms of adaptive resistance of gliomas to a wide range of therapeutics. The same study found an upregulation of the PI3K-AKT pathway in gliomas with CD99 overexpression and a potential sensitivity to inhibitors of this pathway [26].

Overall, these studies highlight a crucial role of CD99 in glioblastoma, particularly in promoting the migration and invasiveness of cancer cells, though an effect on regulated cell deaths was also reported. CD99 might therefore be a relevant therapeutic target, particularly in counteracting the typical tendency of glioblastoma cells to disseminate widely throughout the brain tissue. Given these premises, our finding of a higher CD99 expression in tumors undergoing complete resection than in those undergoing partial resection or biopsy cannot be easily interpreted. A possible explanation is a chance finding related to the low number of patients in our study. However, the expression of CD99 varies in the various subtypes of glioblastoma [23] and in different glioblastoma cell lines [21,22], and its biological impact, which has been studied mainly on individual cell lines, could also be variable, as occurs among different types of neoplasms [18].

The lack of a prognostic impact does not necessarily exclude a potential role of CD99 as therapeutic target in tumors with CD99 overexpression. We found no or very weak expression of CD99 in single cells in normal brain tissue. This represents a prerequisite for the potential utility of CD99 as a therapeutic target in those glioblastomas that overexpress this molecule. In preclinical studies, monoclonal antibodies targeting CD99 have shown promise in various cancer types. In Ewing sarcoma, they hinder tumor growth through methuosis, a unique non-apoptotic form of cell death characterized by micropinocytosis [36,37]. They can induce heat shock protein 70 in B-cell precursor acute lymphoblastic leukemia (ALL), promoting natural killer cell-mediated tumor lysis [38]. In acute myeloid leukemia (AML), they induce cell death and inhibit xenografts without the involvement of immune effector cells, via SRC family kinase activation [39]. Notably, AML with FLT3 internal tandem duplication mutations displays strong apoptotic activity from anti-CD99 antibodies, involving the intrinsic and extrinsic apoptotic pathways [29]. Additionally, these antibodies prove effective in mantle B-cell lymphoma [40].

In addition to monoclonal antibodies, several other innovative strategies have been explored to target CD99 in preclinical studies. These include a human IgG-based tetravalent anti-CD99 antibody [41], or a branched multi-peptide composed of ERBB2, BIRC5, and CD99 to generate alpha-type 1 polarized dendritic cells, thereby stimulating cytotoxic T-lymphocyte to exert cytotoxic activity against glioblastoma cell lines and primary cultures [42]. Another approach involved a single-chain variable fragment anti-CD99 antibody conjugated with an elastin-like polypeptide to form nanoworms [43]. Additionally, anti-CD99 chimeric antigen receptor (CAR) T cells, expressing a low-affinity anti-CD99 antibody to avoid targeting normal blood cells, were explored as a potential therapeutic approach in ALL [44]. The same strategy could likely be used for the development of CD99-targeted antibody-drug conjugates in tumors with CD99 overexpression [21,22,23].

Some side effects are certainly expected from anti-CD99 treatments. Based on the HPA data, it is reasonable to expect potential myelotoxicity, immune, gonadal, gastroenteric, and metabolic toxicity [16]. Nevertheless, some preclinical studies show selectivity for cancer cells compared with normal CD99-expressing cells [37,41], and the use of low affinity antibodies has been shown to be effective in leukemic models while sparing normal bone marrow cells [44]. The toxicity of anti-CD99 agents will need to be carefully evaluated in preclinical and clinical studies.

These preclinical studies collectively highlight the diverse and promising ways in which CD99 can be targeted for cancer treatment. Their findings suggest that CD99-directed therapies hold significant potential for effectively combating various types of cancers, and further exploration of these strategies in clinical studies is warranted.

Our study has limitations, mainly related to the sample size and the study’s retrospective nature. Moreover, molecular heterogeneity of the samples could not be explored in our facilities. However, the expression and prognostic impact of CD99 were evaluated by two separate methods, both of which hold potential for clinical use. Furthermore, patients were consecutively and homogeneously treated at our institution, avoiding selection bias and heterogeneity in clinical management. Finally, it should be noted that a cohort of 46 glioblastoma patients for a single-center analysis should not be dismissed, given the rarity of the disease.

The analysis of the TCGA casuistry leads to the same results found in our case study. However, it must be emphasized that the method of evaluation of CD99 expression in the TCGA study (mRNA sequencing) is different from that we adopted. In addition, there are certainly partial differences in the patient populations and treatments received. Although these differences prevent a direct comparison between the two case series, the absence of a prognostic impact of CD99 in such different case series may partially strengthen this result. This does not exclude the necessity for additional confirmatory studies on larger case series.

In summary, our research shows overexpression of CD99 in 22% of glioblastoma samples and lower expression in further 61% of the cases. It confirms that the full-length, wild-type isoform of CD99 is exclusively expressed in glioblastoma. The expression levels measured using qRT-PCR showed a positive correlation with the semi-quantitative evaluation of CD99 expression by IHC. However, CD99 expression levels do not seem to be significantly associated with patients’ prognosis, regardless of whether they were assessed using qRT-PCR or IHC.

Despite not finding a direct association with prognosis, the fact that CD99 is detectable in most glioblastoma cases, along with the preclinical evidence supporting its role in glioblastoma pathogenesis and progression, suggests that it could still serve as a potential therapeutic target. Further investigations are needed to better understand the function of CD99 in different glioblastoma subtypes, its interactions with other relevant biomarkers, and its involvement in the response to various treatments. Further efforts should explore the efficacy of anti-CD99 drugs and identify potential predictors of response through preclinical and clinical studies. Given its frequent expression in glioblastoma, CD99 might be a potential target for pure anti-CD99 antibodies or, in the new era of ADCs with novel anti-CD99, combined with temozolomide.

In conclusion, our study provides further evidence on the expression and potential significance of CD99 in glioblastoma. While not directly linked to overall prognosis in our cohort, CD99 remains a potential therapeutic target and the efficacy of CD99-targeted treatments should be explored.

## Figures and Tables

**Table 1:** Characteristics and univariate analysis of the 1-year overall survival of glioblastoma patients.

Variables	Patients	12-Month Overall Survival	
	N (%)	OS (%)	*p* Value ^a^
Sex			
Male	27 (58.7%)	34.6	0.814
Female	19 (41.3%)	38.9	
Age			
<65	22 (47.8%)	50.0	0.094
≥65	24 (52.2%)	25.0	
Tumor			
Primary	41 (89.1%)	35.9	0.177
Relapse	5 (10.9%)	40.0	
Type of Resection			
Complete resection	11 (23.9%)	50.0	**0.057**
Partial resection	14 (30.4%)	53.8	
Biopsy	15 (32.6%)	20.0	
Not Available	6 (13.0%)		
MGMT promoter			
Methylated	17 (37.0%)	37.5	0.557
Unmethylated	26 (56.5%)	36.0	
Not Available	3 (6.5%)		
Multifocality			
No	38 (82.6%)	38.9	0.467
Yes	8 (17.4%)	25.0	
CD99 IHC			
Negative (0)	8 (17.4%)	50.0	0.734
1+	8 (17.4%)	28.6	
2+	20 (43.5%)	31.0	
3+	10 (21.7%)	40.0	
CD99 qRT-PCR ^b^			
Low	23 (50.0%)	31.8	0.607
High	23 (50.0%)	40.9	

Legend. OS: overall survival. ^a^ Referred to log-rank test. Significant *p* values are highlighted in bold. ^b^ Low and high according to the cut-off median value.

**Table 2:** Multivariable analysis of prognostic factors in glioblastoma patients.

Cox Model: CD99 IHC Expression			Cox Model: CD99 qRT-PCR Expression		
Variable	HR (95%CI)	*p* Value	Variable	HR (95% CI)	*p* Value
Age <65 ≥65	1.00 (Reference) 2.19 (1.03–4.65)	**0.042**	Age <65 ≥65	1.00 (Reference) 2.16 (0.98–4.77)	**0.054**
Resection ^a^ Biopsy Partial Complete	1.00 (Reference) 0.36 (0.14–0.88) 0.36 (0.14–0.95)	**0.024** **0.039**	Resection ^b^ Biopsy Partial Complete	1.00 (Reference) 0.34 (0.14–0.85) 0.38 (0.14–1.02)	**0.021** **0.054**
MGMT promoter Unmethylated Methylated	1.00 (Reference) 1.02 (0.46–2.28)	0.949	MGMT promoter Unmethylated Methylated	1.00 (Reference) 1.15 (0.53–2.48)	0.730
CD99 0 1+ 2+ 3+	1.00 (Reference) 0.83 (0.25–2.08) 0.84 (0.30–2.30) 1.58 (0.50–4.96)	0.770 0.730 0.437	CD99 ^c^	1.04 (0.93–1.15)	0.542

Legend. HR: hazard ratio; CI: confidence interval; IHC: immunohistochemistry; qRT-PCR: quantitative Real-Time Polymerase Chain Reaction. Significant *p* values are highlighted in bold. ^a^ Overall *p*-value: 0.047. ^b^ Overall *p*-value: 0.049. ^c^ Considered as continuous variable.

## Data Availability

The original contributions presented in this study are all enclosed in the article. Further inquiries can be directed to the corresponding author.
